# Multiple Antibody‐Coated Gold Nanoparticle‐Based ExoAssay for Rapid Isolation of CNS‐Specific Exosomes From Blood

**DOI:** 10.1111/jnc.70263

**Published:** 2025-10-28

**Authors:** Leticia Camila Fernandez Flores, Neelam Younas, Stefan Goebel, Kathrin Dittmar, Tayyaba Saleem, Abrar Younas, Holger Budde, Tobias J. Legler, Wiebke Möbius, Peter Hermann, Matthias Schmitz, Inga Zerr

**Affiliations:** ^1^ Department of Neurology University Medical Center Göttingen, National Reference Center for Surveillance of TSE Göttingen Germany; ^2^ German Center for Neurodegenerative Diseases (DZNE) Göttingen Germany; ^3^ Department of Transfusion Medicine University Medical Center Göttingen Göttingen Germany; ^4^ Electron Microscopy Unit, Department of Neurogenetics Max‐Planck‐Institute for Multidisciplinary Sciences Göttingen Germany

**Keywords:** Alzheimer's disease, biomarkers, ExoAssay, neuronal EVs, plasma, tau

## Abstract

In neurodegenerative diseases, brain‐derived extracellular vesicles (EVs)/exosomes from blood offer a great opportunity to explore their contents for their utility as biomarkers. However, the conventional methodologies for the purification of EVs from complex biofluids have many limitations, restricting their clinical implementation. We aimed to optimize a direct, less time‐consuming, affordable, and reliable nanowire‐based method to isolate neuronal EVs from blood plasma. Here, we improved a simple and direct methodology using multiple antibody‐coated magnetic nanowires for efficient and rapid isolation of neuronal EVs (ExoAssay) from human plasma. We characterized the isolated EVs and validated the protocol using multiple approaches, for example, nanoparticle tracking analysis (NTA), immunoblotting, and transmission electron microscopy (TEM). We purified round‐shaped EVs with an average size of 116 nm. We identified the general markers of EVs including CD9, CD63, CD81, and Flotillin‐1 and two neuronal EV markers L1‐cell adhesion molecule (L1CAM) and neural cell adhesion molecule (NCAM) via immunoblotting. Interestingly, the levels of T‐Tau and P‐Tau were upregulated in EVs isolated from Alzheimer's patients (*n* = 30), in comparison with healthy controls. Furthermore, there were no significant differences between CSF‐ and EV‐based Tau levels. The high‐throughput mass‐spectrometry analysis of isolated EVs revealed 280 proteins as significantly modified in Alzheimer's disease cases in comparison with controls. The presented nanotechnology‐based methodology offers an innovative and efficient tool for EV‐based biomarker investigations and clinical utility by simplifying the enrichment of CNS‐originated exosomes from complex biological fluids. This methodology opens up the avenue for longitudinal monitoring of important disease‐related proteins in the brain by analysis of brain‐derived EVs from blood plasma using simple blood withdrawal.

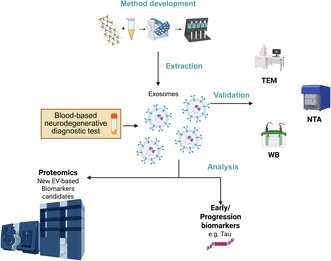

## Graphical abstract

A streamlined nanowire‐based approach (ExoAssay) enabled direct, rapid, and cost‐effective isolation of neuronal extracellular vesicles (EVs) from human plasma using multiple antibody‐coated magnetic nanowires. The isolated EVs were characterized using nanoparticle tracking analysis (NTA), immunoblotting, and transmission electron microscopy (TEM). Proteomic profiling and ELISA assays recapitulated the pathological changes occurring in Alzheimer's disease. The presented assay offers an efficient tool for EV‐based biomarker investigations and clinical utility by simplifying the enrichment of CNS‐originated EVs from complex biological fluids. This methodology opens up the avenue for longitudinal monitoring of disease by analysis of CNS‐EVs using simple blood withdrawal.

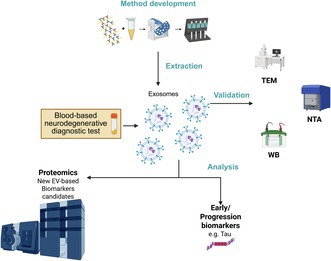

**Abbreviations**
°C: degree celsius
A: ampere
Abs_MNWs: antibody‐conjugated magnetic nanowires
ACN: acetonitrile
AD: Alzheimer's disease
APOE: apolipoprotein E
Ca^++^
: calcium
CD9: CD9 molecule
CD171/L1CAM: L1 cell adhesion molecule
CD81: CD81 molecule
CD63: CD63 molecule
CD56/ NCAM1: neural cell adhesion molecule 1
CSF: cerebrospinal fluid
cm: centimeter
CNS: central nervous system
ddH_2_O: double distilled Water
DIA: data independent acquisition
DTT: dithiothreitol
ELISA: enzyme‐linked immunosorbent assay
EVs: extracellular vesicles
FA: formic acid
FDR: false discovery rate
Gly: glycine
h: hour
HC: healthy controls
IAA: iodoacetamide
IP: immunoprecipitation
ISEV: International Society of Extracellular Vesicles
kDa: kilodalton
L: liters
L.O: left‐over
M: molar
Min: minutes
mL: mililiter
mM: millimolar
MNWs: magnetic nanowires
MRI: magnetic resonance imaging
ng: nanogram
nm: nanometer
NPS: nanoparticles
NTA: nanoparticle tracking analysis
PBS: phosphate‐buffered saline
PBS‐T: phosphate‐buffered saline with Tween 20
PET/PET‐CT: Positron Emission Tomography/Positron Emission Tomography Computer Tomography
%: percent
PrP: prion protein
RRIDs: research resource identifiers
P‐Tau T181: phosphorylated tau threonine 181
PVDF: polyvinylidene difluoride
rpm: revolutions per minute
SDS‐PAGE: sodium dodecyl sulfate‐polyacrylamide gel electrophoresis
sec: seconds
SFPQ: splicing factor, proline‐ and glutamine‐rich
RT: room temperature
Tau: microtubule‐associated protein tau
T‐Tau: total tau
TBS‐T: tris‐buffered saline with Tween20
TEM: transmission electron microscopy
TFA: triflouroacetic acid
TSE: transmissible spongiform encephalopathies
μL: microliters
μg: microgram
V: volt (voltage)
Xg: relative centrifugal force
w/o: without
Aβ 1–40: amyloid‐beta precursor protein 1–40
Aβ 1–42: amyloid‐beta precursor protein 1–42

## 1 Introduction

Dementia is a growing public health problem. By the year 2025, more than 131 million patients will be diagnosed with dementia (Martin Prince et al. 2015). Patients suffering from dementia are dealing with neurodegenerative processes, which lead to progressive and irreversible loss of cognitive functions (Dar et al. 2020). Alzheimer's disease (AD) is one of the most common causes of dementia. The illness duration is approximately 5–10 years (Armstrong 2014), and its clinical diagnosis is defined by the clinical presentation and the detection of disease‐specific biomarkers (phosphorylated Tau and Amyloid‐beta) in the cerebral spinal fluid (CSF) among other tests such as neuropsychology, brain, and amyloid imaging (on positron emission tomography (PET)) (Dubois et al. 2021). Some of these approaches have been shown to identify pathology very early. However, these tests are invasive (lumbar puncture) or expensive and available in specialized centers (imaging) only. The blood‐based biomarkers are emerging (Staffaroni et al. 2019). Since an early diagnosis of AD could help to establish new therapy approaches, there is a huge urge to obtain biomarkers from easily accessible biofluids, for example, blood. Hence, the isolation of extracellular vesicles (EVs) of neuronal origin from blood has gained a lot of interest.

Extracellular vesicles are nano‐vesicles naturally released from the cell. Exosomes are the smallest extracellular vesicles, with a size of ∼30–150 nm (Théry et al. 2018). Exosomes are released from many different types of cells and can be found in almost all body fluids (Théry et al. 2002), including cerebrospinal fluid (Muraoka et al. 2019), blood (Wu et al. 2017), urine (Street et al. 2017), and saliva (Han et al. 2018). Exosomes protect their cargoes by lipid bilayer from degradation and denaturation in the extracellular environments (Iranifar et al. 2019). Since they are present in several biofluids, and their cargos are protected by lipid bilayer, they are receiving much attention as potential biomarkers and drug delivery tools for the diagnosis and treatment of neurodegenerative diseases (Kalluri and LeBleu 2020). Essentially, all cell types release exosomes, including neurons (Watson et al. 2019), and these can cross the blood–brain barrier (Betzer et al. 2017; Lai et al. 2014).

In neurodegenerative disease, attempts have been made to collect neuronal‐derived exosomes from blood using a kit named “ExoQuick” in combination with immunoprecipitation using L1CAM antibody (Fiandaca et al. 2014; Goetzl et al. 2015; Mustapic et al. 2017). The L1CAM neuronal‐derived exosomes from plasma showed high levels of total tau, its phosphorylated form (T181, S396), Aβ1‐42, and hemoglobin in AD patients compared with controls (Fiandaca et al. 2014; Ibrahim Arioz et al. 2021). However, the use of L1CAM as a neuronal marker is controversial. It was shown that L1CAM in the human plasma also exists in free forms (Norman et al. 2021). Therefore, NCAM has also been used as an alternative option as a neuronal exosome marker (Jia et al. 2019).

The emerging studies are showing a great potential for the utility of EV‐based early diagnostic approaches. However, the simplicity, standardization, and diagnostic feasibility of isolation methods for EVs are still lacking. Currently, the majority of the techniques to isolate EVs, specifically exosomes, need expensive equipment; they are time‐consuming and labour‐intensive, lacking implementation for thousands of samples for diagnostic purposes. The implementation of a protocol for clinical applications is challenging, which requires an urgent solution taking into account the quality of the results, simplicity, and low cost (N. Younas et al. 2022).

Nanotechnology approaches have lately emerged as diagnostic tools for future cancer research (Hong et al. 2016; Lee et al. 2016). Several EV isolation methods have been published in the past few years, including a methodology based on a multiple‐antibody coated nanowires protocol that enhanced the capture of cell‐specific EVs from complex biofluids (Lim et al. 2019). Due to the rarity of neuronal EVs in the blood, the yield is quite low with the typical immunoaffinity approaches. The advantages of a nanowires‐based protocol to isolate EVs by targeting their surface markers include the identification of EVs with phenotypic variation, reduction of the loss of circulating exosomes during the isolation, flexibility of antibody conjugation, its large surface area due to its long and thin morphology, and its cost‐effectiveness.

Accordingly, we aimed to optimize a direct, less time‐consuming, affordable, and reliable nanowire‐based method to isolate neuronal exosomes from blood plasma. For this, we coated the magnetic nanowires with two neuronal markers (L1CAM, NCAM) and one exosome membrane marker (CD81) and investigated their potential to measure traditional neurodegenerative biomarkers.

## 2 Materials and Methods

### 2.1 Ethics Statement

The following study was planned and carried out at the National Reference Centre for TSE and Dementia research group, Department of Neurology at the University Medical Centre Goettingen. The ethic commission of the Medicine Faculty of the Georg August University certified the ethic approval for this work (Reference number: 9/6/08). All samples were analyzed blindly for at least personal data.

### 2.2 Patient Cohorts and Sample Processing

The samples from Alzheimer's disease patients were collected from the National Reference Center for Transmissible Spongiform Encephalopathies as previously described (Hermann et al. 2021; Llorens et al. 2020). The healthy control (HC) samples were obtained from the Department of Transfusion Medicine at the University Medical Centre Goettingen, based on fulfilment of the official blood donor criteria (Reference number: 2/7/2021). Eligible individuals must meet the following criteria: they must be free of chronic illnesses, have no permanent regime of medication, be over 18 years old, and weigh more than 50 kg. If any medication is used, a physician evaluates the specific case for eligibility. Informed consent was obtained from all participants. According to the availability of plasma samples and clinical information, *n* = 30 samples were analyzed. No formal sample size calculations were performed a priori. The number of subjects included was determined based on logistical considerations and the availability of eligible participants during the study period. Many published studies in the EV biomarker field (Anastasi et al. 2021; Li et al. 2020; Lim et al. 2019), especially at the discovery and pilot stage, used sample sizes in a similar range and reported sufficient statistical power (Anastasi et al. 2021; Li et al. 2020; Lim et al. 2019; Zhao et al. 2019).

### 2.3 Nanowires EV Isolation Protocol

**FIGURE 1:**
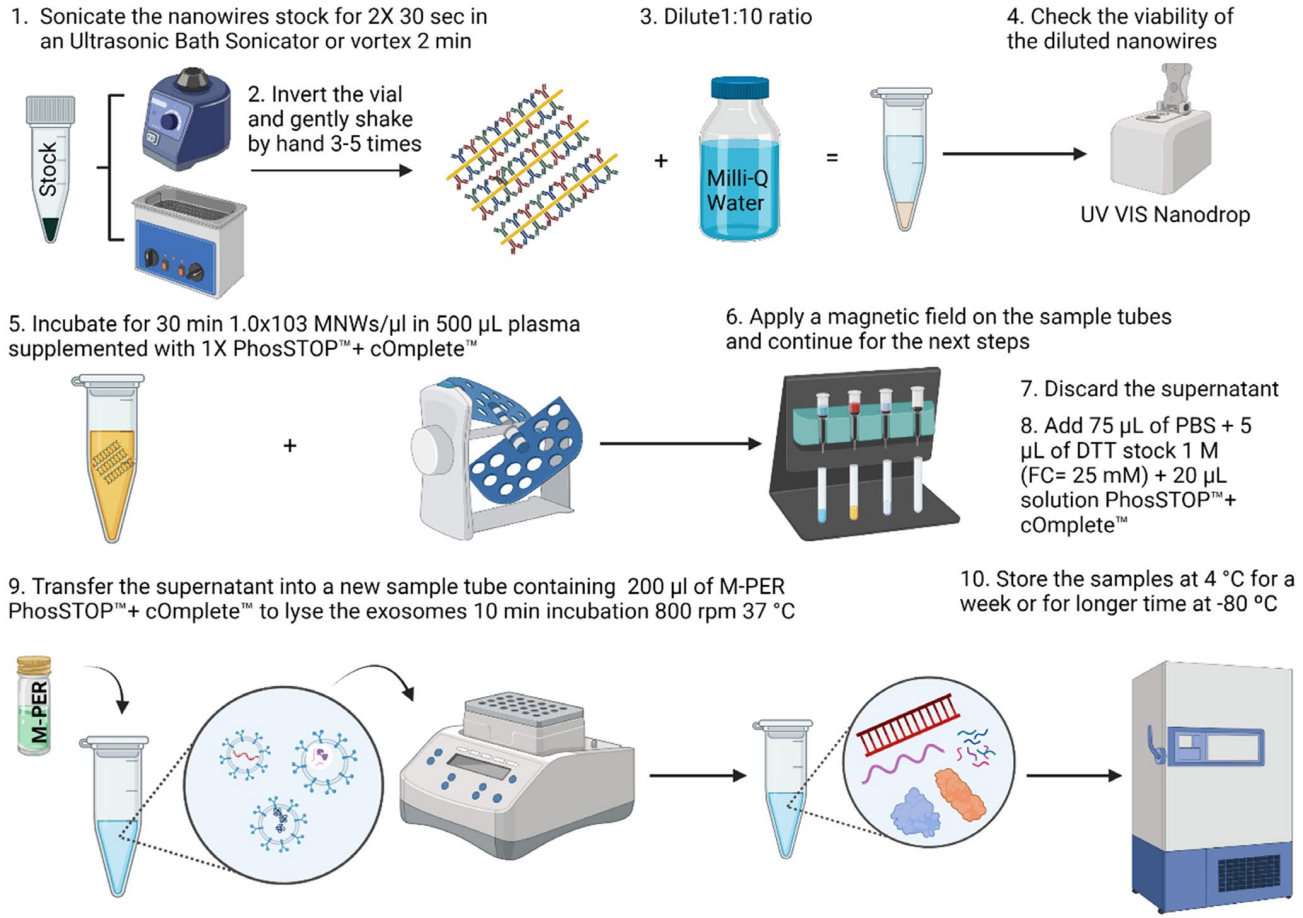
Neuronal EV isolation final protocol flow chart. Here is shown a scheme of the steps to isolate cell‐specific EVs from human plasma samples. It is a rapid, cost‐effective, and easy protocol to follow. Created in Biorender (RRID: SCR_018361).

Whole blood was centrifuged at 2000 × g for 10 min at 4°C. The layer of plasma was transferred to a tube (Catalog #E1420‐2340) and immediately frozen at −80°C in separate aliquots. The plasma samples without signs of hemolysis were carefully taken in order to avoid contamination from blood cells. Plasma (0.5 mL) supplemented with protease and phosphate inhibitors [Merck (RRID: SCR_001287)] was centrifuged at 3000 × g for 15 min at 4°C to remove cellular debris and cells. The supernatant was collected in new tubes followed by another centrifugation at 10000 × g for 20 min. After this centrifugation, the supernatant was transferred to new 1.5 mL tubes. Neuronal‐derived EVs were isolated using antibody‐conjugated magnetic nanowires (Abs_MNWs, NanopartzTM Cat. No. #CM4‐10 k‐CUSTOM‐DIH). We conjugated the magnetic nanowires with two neuronal EV markers (L1CAM and NCAM) and a general exosomal surface marker (CD81) with a final antibody concentration of 10 μg/mL. Concisely, 0.5 mL plasma [supplemented with phosphatase inhibitors and protease inhibitors; Merck (RRID: SCR_001287)] was incubated with Abs_MNWs (1.0 × 10^3^ MNWs/μL) for 30 min at room temperature with gentle shaking. To remove the supernatant from the sample, a magnetic field was applied to the 1.5 mL tubes. DTT (Sigma‐Aldrich, RRID: SCR_008988 Cat. No. 10708984001) was added at a final concentration of 25 mM to release the EVs from the nanowires as reported previously (Lim et al. 2019). Both the elution buffer and the lysis buffer were supplemented with phosphatase and protease inhibitors (Figure 1). We also added PhosSTOP + cOmplete to all the steps of the protocol (Kapogiannis et al. 2015).

### 2.4 Nanoparticle Tracking Analysis (NTA)

The size and number of the EVs were determined using nanoparticle tracking analysis. The NTA was performed using a NanoSight LM10 instrument, an LM14 viewing unit equipped with a 532 nm laser and NTA 2.3 software (NanoSight, Salisbury, UK). For the analysis, the samples were diluted in PBS (1×) to a final volume of 1000 μL. The recording characteristics were as follows: detection threshold of five, camera level of 14 at 25°C, and videos of 3 × 60 s.

### 2.5 Transmission Electron Microscopy (TEM)

To see the morphology of isolated EVs, we performed Transmission Electron Microscopy using negative staining as described (Théry et al. 2006). Briefly, TEM copper grids (150 hexagonal mesh, Science Services, Germany) with a formvar film were floated onto a 10 μL suspension of the EV fraction for 10 min. Afterwards, PBS was used to wash the grids (5×). For contrast development, the grids were incubated on an aqueous solution of uranylacetate‐oxalate for 5 min, followed by a 5 min incubation on droplets of a (10‐time dilution) of 4% uranyl acetate in 2% methylcellulose. The methylcellulose was blotted from the grids using filter paper and dried of the methylcellulose film. For image acquisition, a LEO912 transmission electron microscope [Carl Zeiss Microscopy, Germany Zeiss Microscopy (RRID:SCR_023607)].

### 2.6 SDS‐PAGE Electrophoresis

Lysates of the samples or plasma samples were denatured using ROTILoad 1 or ROTILoad 2 and cooked for 5 min at 95°C or for 10 min at 70°C [Roth Laboratory (RRID:SCR_005711) Cat. No. K929.1 and K930.1]. Next, they were loaded onto a 12% homemade Tris/glycine gel and subjected to electrophoresis for ~2 h (20 min with 80 V, 30 min with 120 V, and 60 min with 200 V) using Tris‐Gly running buffer. For eluates, 30 μL was run in each gel. For input plasma and leftover fraction, 10% volume of the eluates was run in each gel.

### 2.7 Coomassie Staining

The Coomassie staining was performed as published (Eraña et al. 2019). Concisely, when the SDS‐PAGE electrophoresis was completed, the gels were washed with ddH2O and stained with BlueSafe Coomassie stain (NZYTech (RRID:SCR_016772) Cat. No. MB15201) for 2 h at RT with gentle shaking. Destaining of the gel was reached with several ddH2O washes for ~1 h with gentle shaking.

### 2.8 Western Blotting

After SDS‐PAGE gel electrophoresis, gels were transferred onto an activated polyvinylidene difluoride membrane with a 0.45 μm pore size using a Trans‐Blot SD Semidry blot chamber (Bio‐Rad, Hercules, USA) for 70 min at 16 V with 1.5 A. The activation of the 6.5 × 8.5 cm PVDF membrane was done with 1 min of pure methanol and 1 min in Transblot buffer. Subsequently, the blocking step was carried out for 1 h at RT using blocking buffer with gentle shaking. The membranes were probed with the antibodies overnight at 4°C with shaking. Afterward, the membranes were washed with TBS‐T and incubated for 1 h with a secondary antibody coupled to horseradish peroxidase. Finally, the membranes were developed using the enhanced chemiluminescent method with Chemi‐Doc [Bio‐Rad Laboratories (RRID:SCR_008426)]. Antibodies: NCAM‐L1 (Abcam Cat# ab24345, RRID:AB_448025); NCAM (Santa Cruz Biotechnology Cat# sc‐7326, RRID:AB_627127); CD‐81 (Santa Cruz Biotechnology Cat# sc‐23 962, RRID:AB_627192); TSG 101 (Santa Cruz Biotechnology Cat# sc‐7964, RRID:AB_671392); Alix (Cell Signaling Technology Cat# 2171, RRID:AB_2299455); CD9 (Cell Signaling Technology Cat# 13174, RRID:AB_2798139); Flotillin‐1 (Cell Signaling Technology Cat# 18634, RRID:AB_2773040).

### 2.9 Enzyme‐Linked Immunosorbent Assay (ELISA)

Detection of two common biomarkers [T‐Tau and P‐Tau (181P)] was accomplished in neuronal‐derived EVs and cerebrospinal fluid using conventional ELISA. The profiles of study participants (*n* = 30) are detailed in Table 1 and Table S1.

– Total Tau: INNOTEST hTAU (Cat. No. FR56730 FUJIREBIO).
– P‐Tau (181P): INNOTEST PHOSPHO‐TAU (Cat. No. FRI52215 FUJIREBIO).

**TABLE 1:** Pathological profiles of the AD patients.

No.	Sex	Age	MMSE	APOE	CSF T‐Tau (pg/mL)	CSF P‐Tau (pg/mL)	CSF Aβ 1–40 (pg/mL)	CSF Aβ 1–42 (pg/mL)	Disease stage
1	M	82	24	3/4	121	34	9158	858	Mild
2	M	73	5	3/4	279	42	7803	120	Severe
3	F	72	13	3/4	1007	149	10 334	433	Severe
4	M	86	18	3/4	317	40	5500	446	Severe
5	F	85	24	3/3	670	48	8340	496	Mild
6	F	77	16	3/4	209	54	11 534	718	Moderate
7	F	56	16	3/4	840	64	4082	473	Moderate
8	F	82	22	3/4	439	67	9690	691	Mild
9	F	66	23	3/4	372	69	16 061	855	Mild
10	M	65	27	3/3	847	70	10 162	1389	Mild
11	F	64	17	3/4	847	70	10 162	1389	Moderate
12	F	72	11	3/4	268	76	2903	439	Severe
13	M	53	7	3/4	547	76	9861	526	Severe
14	F	79	24	3/3	652	83	14 068	570	Mild
15	M	81	15	3/3	747	83	8073	456	Severe
16	M	65	25	2/3	565	88	13 448	683	Mild
17	M	69	20	3/4	933	94	11 910	582	Moderate
18	F	77	20	3/4	< 75	94	7844	478	Moderate
19	M	63	24	3/4	703	111	6786	476	Mild
20	F	73	13	4/4	608	128	3447	161	Severe

*Note:* Disease severity was determined by the Mini‐mental state examination (MMSE) score (> 25: no cognitive impairment, 25–20: mild cognitive impairment, 20–15: moderate cognitive impairment, < 15: severe cognitive impairment).

Abbreviations: Aβ, amyloid‐beta; AD, Alzheimer's disease; CSF, cerebrospinal fluid.

### 2.10 Mass Spectrometry Analysis

After the lysis of our isolated EVs, we performed mass spectrometry analysis (biological replicates, *n* = 18, technical replicates *n* = 54) (Table 1 and Table S1). Samples containing neuronal‐derived EVs were processed as described previously (Younas et al. 2024). Briefly, the samples were run on SDS‐PAGE for ~1 cm to obtain a single thick band. After excision from the gel, this band was processed for reduction and alkylation followed by digestion with trypsin (overnight at 37°C). After digestion, the peptides were isolated and concentrated with a speedVac.

For mass spectrometry measurements, the mixture of peptides was concentrated on a Reversed Phase C18 precolumn (0.15 mm ID × 20 mm, self‐packed with Reprosil‐Pur 120 C18‐AQ 3 μm material). It was followed by separation using Reversed Phase C18 nanoflow chromatography on a Picofrit column, 0.075 mm ID × 200 mm (New Objective, Woburn, USA) and a 15 min linear gradient on an Easy nLC‐1000 nanoflow chromatographic system (Thermo Fisher Scientific (RRID:SCR_008452)). For measurement of samples, a Q‐Exactive hybrid quadrupole/orbitrap MS system was used. Spiking was done with Biognosys iRT peptide standard. Quantification and identification by data‐independent acquisition (DIA) on Bruker timsTOF Pro (400 ng equivalent loaded, 40 min gradient, 12 × 2 variable window diaPASEF method, 2 technical replicates/sample). Data processing was performed in Biognosys Spectronaut v16.0.220606.53000 (Hawking) and Perseus. For identification of proteins and spectral library generation from DIA experiments, Pulsar search engine against UniProtKB (RRID: SCR_004426) *Homo sapiens* v12.2021 with default parameters and at a FDR of 1% was used. Data‐independent acquisition quantification was achieved using up to 6 fragments per peptide, up to 10 peptides per protein, dynamic retention time alignment, dynamic mass recalibration, and quartile normalization, at a 1% FDR.

### 2.11 Statistical Analysis

Mass spectrometry data was analyzed in Perseus software for differential expression analysis, and volcano plots were generated in R Project for Statistical Computing (RRID:SCR_001905) (version 3.4.3). After the quantification of protein levels in the different samples, raw values were normalized by log transformations followed by Welch's *t*‐test (*n* = 18, controls: 6, AD cases: 12). For ELISA measurements, the data were tested for normality using the Shapiro‐Wilk normality test. As the data were non‐normal, the Mann–Whitney test was performed. There were no tests used for outliers. Statistical significance was considered at *p* < 0.05 using 95% confidence intervals. Statistical tests were applied using GraphPad Prism 9.01 (RRID: SCR_002798).

## 3 Results

### 3.1 Profiles of the Study Participants

After all participants or their legal representatives were informed and their consent for the analysis of their biological samples was given, we analyzed the clinical data as shown in Table 1 and Table S1.

### 3.2 Characterization of EVs Isolated With the Magnetic Nanowires by NTA and TEM

We evaluated this nanowire‐based protocol for exosome isolation using three different methods according to the recommendations of the International Society for Extracellular Vesicles (Théry et al. 2018) using NTA, immunoblotting, and TEM.

Firstly, to understand the behavior of our customized nanowires, we evaluated the NTA results of each step of the protocol. Original plasma and the nanowires‐bound EV fractions were 1:2000 diluted (due to the high concentration of nanoparticles). The elution fraction was diluted at a ratio of 1:1000. The peaks of different size ranges were observed in each fraction (Figure S1). Firstly, in original plasma samples, a prominent peak of 118 nm was detected; however, 95 and 202 nm nanoparticles also produced small peaks (Figure S1A), suggesting a broad range of nanoparticles in plasma. When plasma was incubated with nanowires, a wider peak was detected with an average size of 130 nm (Figure S1B). The NTA ratified the binding of nanowires to the EVs as shown in Figure 3. Next, the EVs were detached from the nanowires using DTT to obtain the elution fraction. It was observed that the isolated fraction contains EVs in the size range of exosomes (~40–150 nm) (Figure S1C) since the largest peak is 64 nm, followed by 95 nm.

**FIGURE 2:**
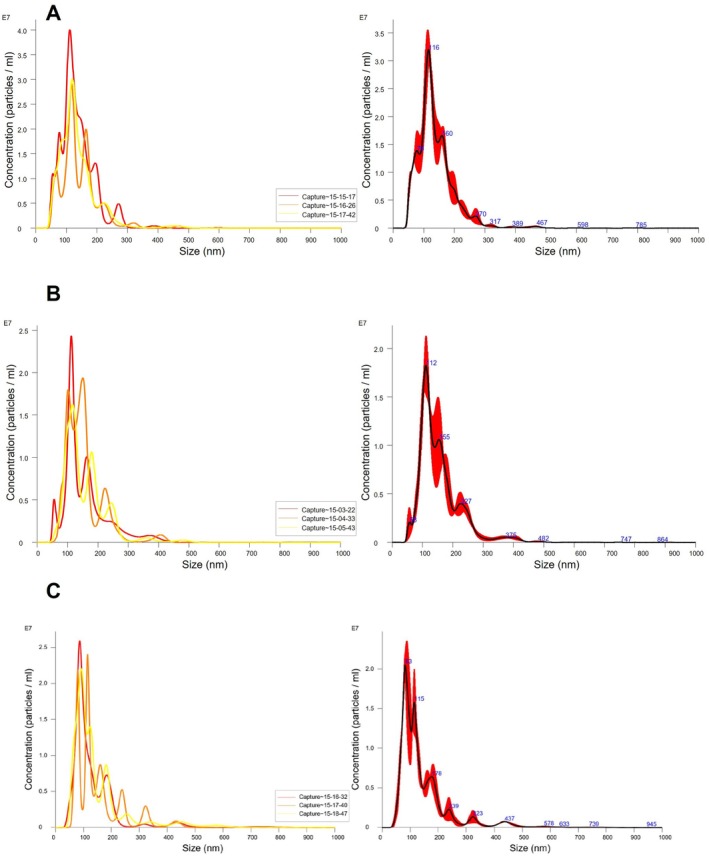
Nanoparticle tracking analysis (NTA) of the eluates in triplicates. (A–C) Representative NTA profiles of EVs showing nanoparticles with prominent peaks of 116, 112, and 83 nm (Error bars indicate ±1 of standard error of the mean) from three independent biological replicates. The left panels display three consecutive measurements (60 s) for each biological replicate. The right panel is showing the average of all three measurements from each replicate.

Then, we performed our nanowire‐based protocol in triplicates and evaluated the eluates by NTA. The L1CAM, NCAM, and CD81 gold‐coated magnetic nanowires successfully isolated nanoparticles in the size range of exosomes according to NTA, as demonstrated in Figure 2A–C. The results demonstrated the excellent performance of our protocol when the EVs were isolated at three different times with three biological replicates (Figure 2A–C). This validation also allowed us to test the reproducibility of our protocol since the size of EVs remains consistent in different experimental replicates (Figure 2).

To visualize EVs' morphology and their size distribution, we used negative staining electron transmission microscopy (TEM). Figure S2 shows the different captures and their expanded images (zoom), with each one representing a different step from the protocol. In all these steps, different sizes and morphologies were observed as expected. The highly packed nanowires (only nanowires fraction) were observed via negative staining (Figure S2A). Additionally, nanowires surrounded by several EVs were observed in separate parts of the capture (EV‐bound nanowire fraction) as shown in Figure S2B. The eluted EVs were found to be randomly distributed, showing diverse size and morphology variations (elution fraction) (Figure S2C). These spheroidal vesicles have diameters smaller than 250 nm.

**FIGURE 3:**
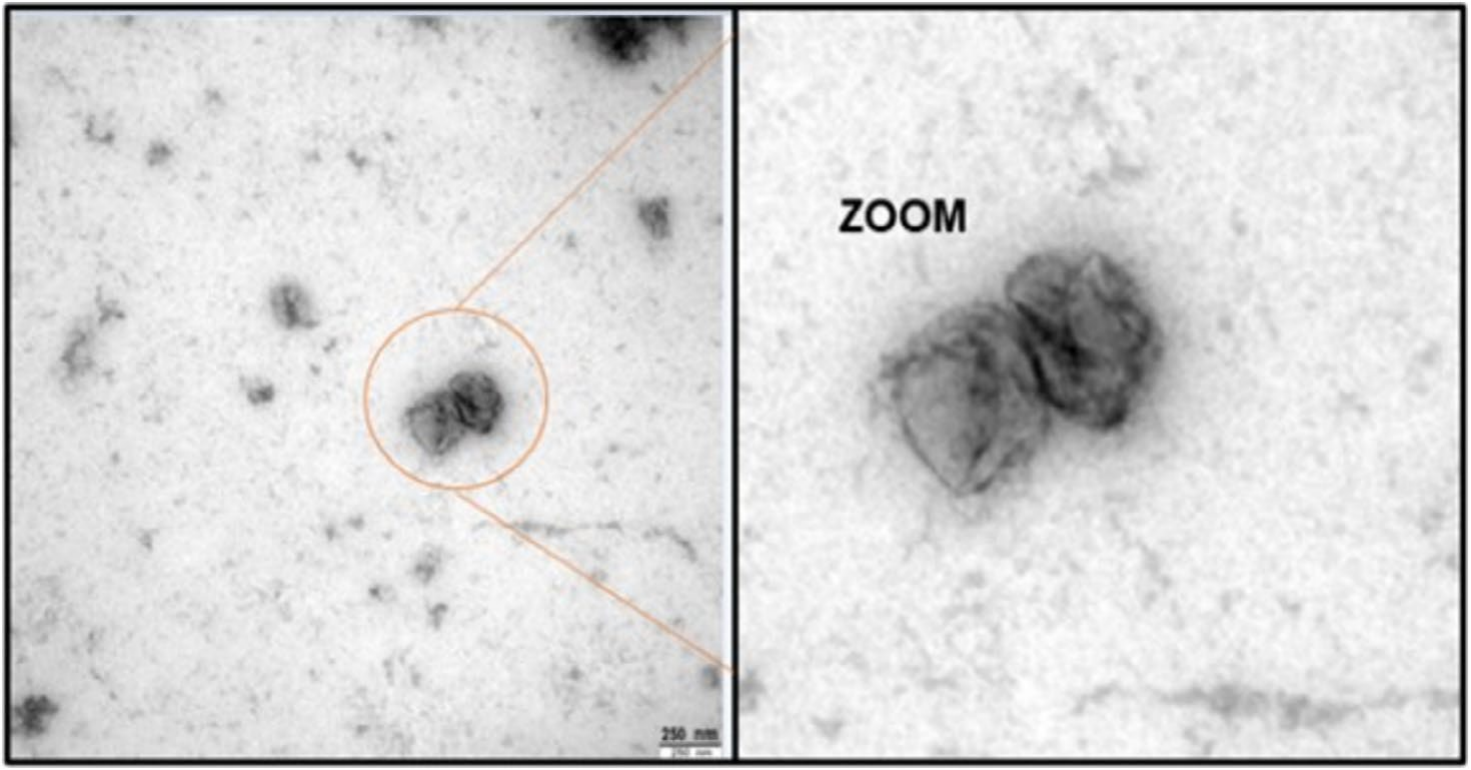
Transmission electron microscopy (TEM) images. Representative TEM image (with expanded zoom) of isolated EVs from blood plasma using antibody‐coated magnetic nanowires. Additional images are in Figure S3. Cup‐shaped vesicular structures were identified, showing the typical morphology of EVs. Bar = 250 nm.

Finally, we performed the characterization of the eluted EVs in triplicates by electron transmission microscopy using negative staining (Figure 3 and Figure S3). This method was followed according to the recommendations of the International Society for Extracellular Vesicles. As shown in Figure 3 and Figure S3, we were able to observe several individual extracellular vesicles. It is also important to mention that in the three images, the shape of the EVs is similar to typical vesicular sac‐like structures in shape (Figure S3).

### 3.3 Characterization of EVs Enriched for Neuronal Origin Using Protein Markers

**FIGURE 4:**
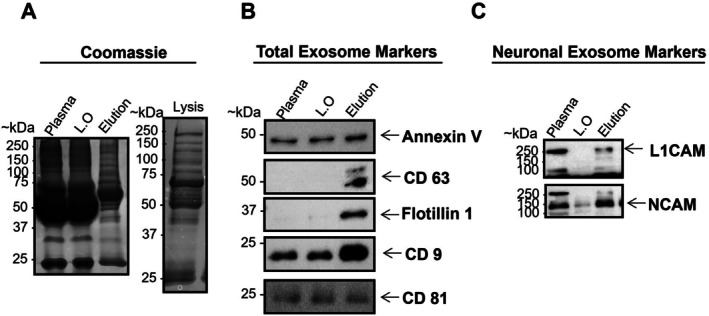
Immunoblotting of the different fractions of the protocol. (A) A gel with Coomassie‐staining displaying all the fractions [input plasma, left over fraction, elution fraction, and lysed eluate (Lysis)] from the nanowire‐based protocol. (B) Immunoblotting of surface and internal exosome markers (CD9, CD63 & CD81, Flotillin 1 & Annexin V). Different proteins were probed on separate membranes, with each membrane probed individually for a specific target protein. (C) Immunoblotting of neuronal EV markers (L1CAM ~200 kDa and NCAM ~140 kDa). Full‐length version and cleavage fragments were identified for L1CAM protein.

Initially, we carried out SDS‐PAGE gel electrophoresis followed by Coomassie staining, which revealed the distinct pattern in each fraction (Figure 4A). Firstly, we could observe all plasma proteins in the first line, followed by a small decrease in intensity in the leftover fraction, as expected. Our elution fraction showed a distinct band pattern. To confirm that our eluates were enriched for extracellular vesicles, we performed immunoblotting analysis to detect EV markers (Figure 4B,C). We identified five different EV markers (CD9, CD63, CD81, Annexin V, and Flotilin‐1). To confirm the neuronal origin of our isolated fractions, we also identified two neuronal markers (L1CAM and NCAM) via immunoblotting. The neuronal markers, L1CAM and NCAM, showed enrichment (higher signal bands) in the elution fraction in comparison with the original plasma, suggesting successful isolation and enrichment of EVs of neuronal origin. It is also important to note the absence of signal in the leftover fractions (Figure 4B,C), indicating successful binding of EVs with the magnetic nanowires.

We also calculated and analyzed this protocol to estimate its suitability for diagnostic purposes. The ExoAssay allowed us to efficiently extract neuronal‐enriched EVs in less than an hour with simplicity and ease, without requirement of any special equipment and complex sample preparations. It is also important to mention that the cost of each sample by this method is ~30 €. All these advantages suggest the utility of this technology could be suitable for diagnostic purposes for neurodegenerative diseases.

### 3.4 Protocol Validation

In order to see if our isolated EV fractions recapitulate the pathological changes occurring in Alzheimer's disease, in terms of dysregulation (upregulated) of total‐Tau and phosphorylated‐Tau (P‐Tau 181), we measured the levels of total‐Tau and its phosphorylated form by ELISA assays in our isolated EV fractions.

### 3.5 Increased Total‐Tau and P‐Tau Levels in Neuronal‐Derived EVs

**FIGURE 5:**
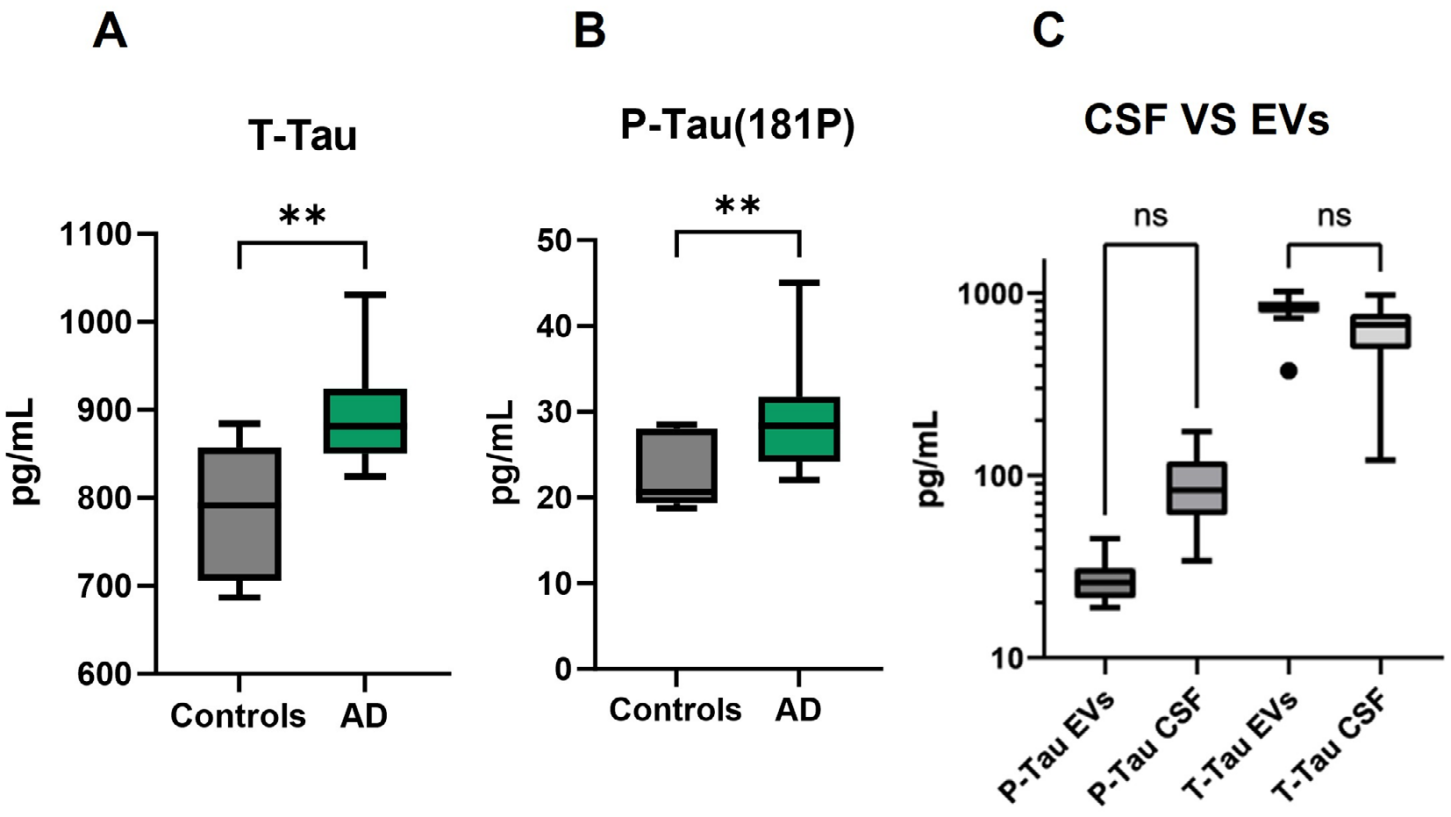
Exosomal levels of Total‐Tau & P‐Tau (181P). T‐Tau and P‐Tau (181P) were measured by ELISA assays (*n* = 23, AD = 14 & Controls = 9). (A) Exosomal T‐Tau from Alzheimer's disease (AD) vs. exosomal T‐Tau from healthy controls (HC) (Mann–Whitney test, *U* = 16, **p = 0.0020). (B) Exosomal P‐Tau from Alzheimer's disease cases (AD) vs. EV P‐Tau from HC (Mann–Whitney test, *U* = 21.50, **p = 0.0072). (C) Total‐Tau and P‐Tau (181P) levels in CSF and EVs were compared from AD patients (*n* = 15). Group differences were evaluated using the Friedman test (statistic = 36.97, df = 3, *p* < 0.0001). Based on our focus on Tau proteins in different compartments, Dunn's post hoc test was applied only for P‐Tau EVs vs. CSF and T‐Tau EVs vs. CSF. No significant difference was observed for either comparison (both *p* > 0.05). Only these pairs were compared, as our principal aim was to evaluate differences in Total‐Tau and phosphorylated‐Tau levels between extracellular vesicles and cerebrospinal fluid. The box represents the interquartile range (IQR) for each group (Controls and AD). The horizontal line inside each box denotes the median value. The whiskers extend from the box edges to the minimum and maximum data values.

Strikingly, there was a significant increase in the levels of T‐Tau and P‐Tau (181P) in exosomes isolated from Alzheimer's disease cases, in comparison with healthy controls (HC), validating our protocol. These results suggest the biomarker potential of neuronal‐derived exosomal fractions to distinguish AD patients from healthy controls (Figure 5A,B). Further, we also compared each patient's CSF concentrations of total‐Tau and phosphorylated‐Tau with corresponding levels detected in blood‐derived EVs. There was no significant difference between CSF and EV‐derived concentrations of total‐Tau and its phosphorylated form (Fiure 5C), suggesting that a less‐invasive blood EV‐based measurement could perform equally well to distinguish diseased cases from controls.

We also compared CSF levels of Total‐Tau and P‐Tau (181P) with our EV‐based levels and found that there were no significant differences between CSF and EV‐based markers. These results suggest the utility of EV‐based analysis of neurodegenerative biomarkers from less invasive samples, for example, blood, in comparison with invasive lumbar puncture for CSF isolation.

Additionally, we were interested in performing a proof of principle to detect neurodegenerative disease‐associated aggregated prone proteins (Prion protein and Tau) in the exosome fractions. Therefore, we performed an immunoblotting analysis to confirm the presence of PrP in EV fractions isolated from healthy controls and prion disease cases (Creutzfeldt‐Jakob disease: CJD) (Figure S4A). The T‐Tau and P‐Tau (S199) were identified in EV‐isolated fractions from healthy controls and AD subjects (Figure S4B). The successful identification of the hallmark proteins (Tau and PrP) in our EVs further strengthens the potential of our nanowire‐based assay for the detection of neurodegenerative disease‐related signatures.

### 3.6 Mass Spectrometry‐Based Potential New Candidates

Finally, we were curious to explore new potential EV‐based biomarkers for AD patients in our EV preparations using quantitative mass spectrometry analysis. For this analysis, EVs isolated from AD cases and healthy controls (biological replicates, *n* = 18 and technical replicates, *n* = 54) were analyzed.

**FIGURE 6:**
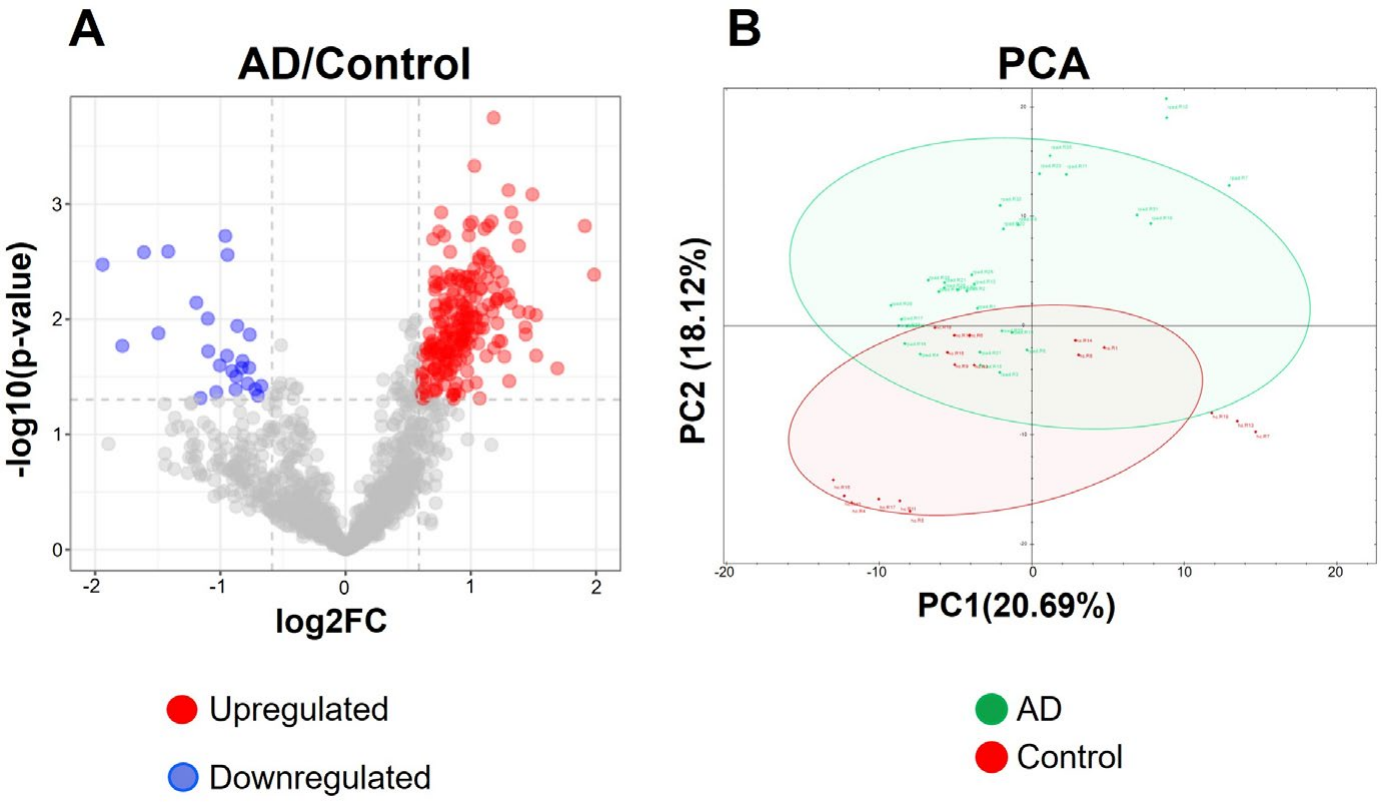
Quantitative mass spectrometry analysis. (A) A volcano plot is showing differentially modified proteins in AD EV fractions (*n* = 12), in comparison with control‐isolated EV fractions (*n* = 6). Each biological replicate was measured in triplicates (technical replicates, *n* = 54). Welch's *t*‐test with a *p*‐value < 0.05 and FC ≥ ±1.5 were considered significantly modified. Red = upregulated protein candidates; blue = downregulated protein candidates. (B) Principle component analysis of proteomic profiles between AD (green) and healthy controls (red). AD, Alzheimer's disease; HC, healthy controls.

The principal component analysis of mass spectrometry data showed a differential proteomic profile in Alzheimer's disease patients (green) in comparison with healthy controls (red) as displayed in Figure 6B. The neuronal EV‐biomarker candidates were uploaded to Perseus software, followed by pairwise comparisons between the two groups using Welch's *t*‐test. Given the exploratory nature of the study, adjustments for multiple comparisons were not applied. The proteins having a *p*‐value < 0.05 and FC ≥ ±1.5 were considered significant hits. There were 246 proteins that were statistically modified using the above criteria in AD EV fractions in comparison with healthy controls (Table S2). A volcano plot is showing the upregulated proteins (red) and the downregulated proteins (blue) (Figure 6A).

The following proteins represented the highest statistical significance: Barrier‐to‐autointegration factor (BANF1), Protein S100‐A6 (S100A6), and Calcium‐activated neutral proteinase small subunit (CAPNS1). A summary of the few significant proteins is displayed in Table 2 with their −log10 *p*‐value and log2 fold change values.

**TABLE 2:** Top ten potential candidates for neuronal exosome biomarkers.

No.	Gene names	Protein description	*p* (−Log 10)	FC (Log2)
1	BANF1	Barrier‐to‐autointegration factor	3.74674	1.18231
2	RBMXL1	RNA‐binding motif protein, X‐linked‐like‐1 (Fragment)	3.32996	1.02872
3	MYBBP1A	Myb‐binding protein 1A	3.11784	1.30073
4	TRIM25	E3 ubiquitin/ISG15 ligase TRIM25	3.08186	1.49135
5	S100A6	Protein S100‐A6	2.92679	1.32309
6	SUPT16H	FACT complex subunit SPT16	2.92619	0.763405
7	RAB10	Ras‐related protein Rab‐10	2.84384	1.00987
8	DUT	Deoxyuridine 5′‐triphosphate nucleotidohydrolase	2.81832	0.988746
9	CAPNS1	Calcium‐activated neutral proteinase small subunit	2.80853	1.91021
10	PSME3	Proteasome activator complex subunit 3	2.79642	1.3577

**FIGURE 7:**
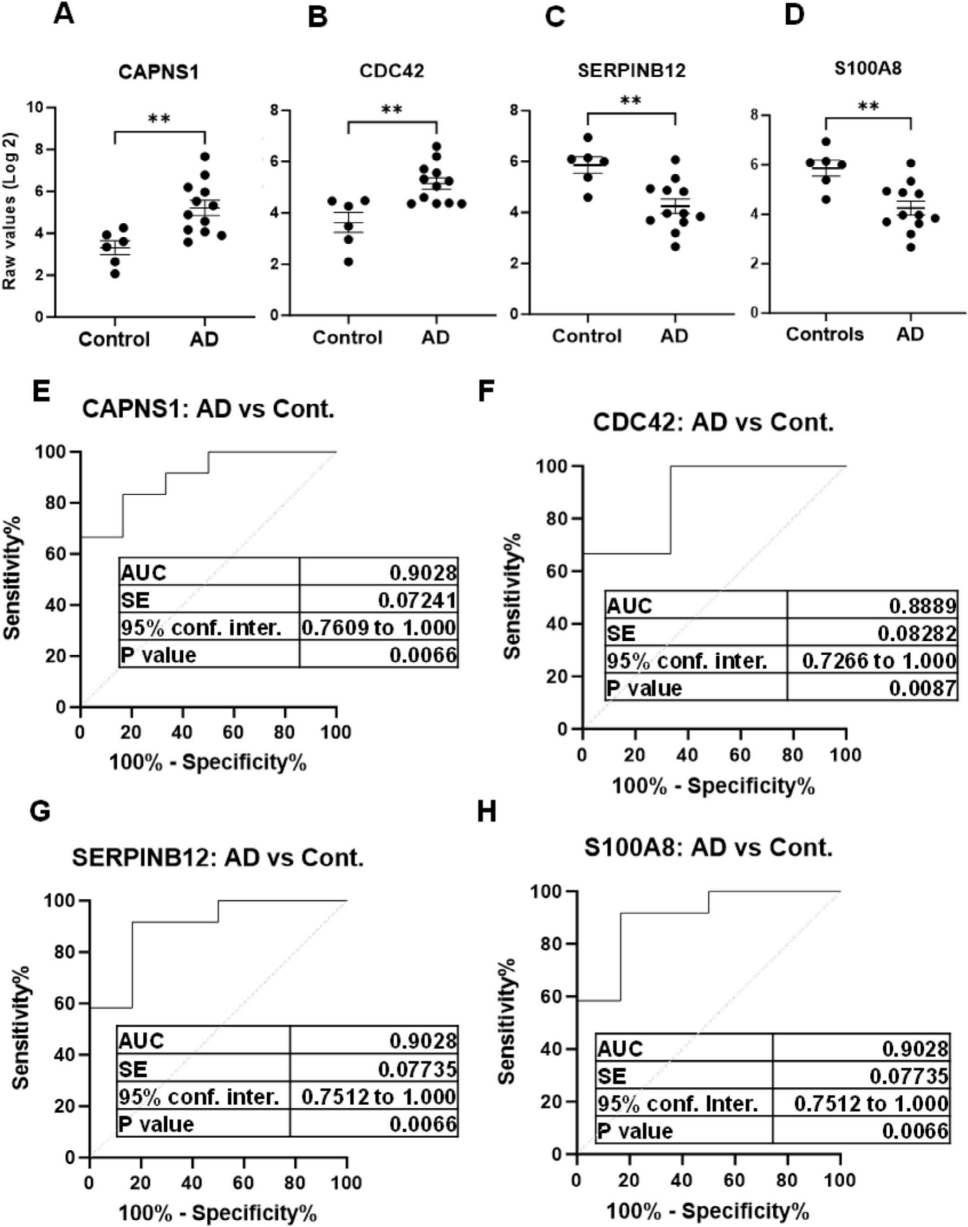
Differentially abundant proteins in Alzheimer's disease EVs. Few examples of significantly abundant protein candidates from mass spectrometry analysis of exosomal fractions of AD cases and healthy controls. The raw intensity values of biological replicates (*n* = 18, technical replicate *n* = 36) from mass spectrometry analysis were used for comparisons [Welch's *t*‐test, healthy controls (*n* = 6) &andAD (*n* = 12)] with significance at *p < 0.05, **p < 0.01. Data is represented as Mean ± SEM. (A) **p = 0.0016, *t* = 3.878, df = 14.60. (B) **p = 0.0092, *t* = 3.373, df = 8.303. (C) **p = 0.0026, *t* = 3.765, df = 12.18. (D) **p = 0.0026, *t* = 3.765, df = 12.18. AUC, area under the ROC curve; Cont., controls; SE, standard error.

Few examples of significantly modified proteins are displayed in Figure 7. The proteins CAPNS1 and CDC42 were significantly more abundant in AD EV fractions, as compared with controls (Figure 7A,B). The proteins SERPINB12 and S100A8 were significantly less abundant in AD‐EV fractions, in comparison with controls (Figure 7C,D).

## 4 Discussion

Purification of cell‐specific extracellular vesicles (EVs) from less‐invasive biological fluids offers a window to look into pathophysiological changes occurring in devastating neurodegenerative diseases. However, the conventional methodologies for the purification of EVs from complex biofluids have many limitations, restricting their clinical implementation. To this end, in the current study, we optimized an easy, less time‐consuming, and diagnostic feasible technique to isolate neuronal‐derived EVs using a magnetic nanowire‐based approach (ExoAssay) (Hong et al. 2016; Lim et al. 2019). To validate our protocol, the requirements from the International Society for Extracellular Vesicles were fulfilled (Théry et al. 2018). The usage of nanowires gives the possibility to conjugate multiple antibodies, and their large surface area offers added advantage to capture small amounts of neuronal EVs from blood plasma. There are controversial findings in the literature about the most used marker (L1CAM) to isolate neuronal extracellular vesicles (Nogueras‐Ortiz et al. 2024; Norman et al. 2021). To overcome this challenge, we developed a methodology that allows flexibility for selection and conjugation of multiple markers instead of relying on a single marker to enrich low‐abundance neuronal EVs from biofluids. Recently, new studies are showing some additional markers that could also be useful, although they need further validations (Eitan et al. 2023). A key advantage of our methodology is its flexibility: our nanowire‐based platform allows for the conjugation of up to five different antibodies, enabling the incorporation of newly validated markers as they become available. Initially, we also conjugated CD81 with the nanowires along with L1CAM and NCAM to maximize the yield. However, future studies should only use neuronal‐specific markers to improve the specificity of cell‐specific EV isolations. To generate a directly comparable negative control (e.g., IgG) for this multi‐antibody approach, we would need to use three corresponding isotype control antibodies in combination. Using three different isotype controls together could potentially capture a broader range of non‐specific components from plasma. This could lead to an overestimation of background binding and make direct comparison with the multi‐antibody capture complicated. However, in future developments, different additional controls for comparison should be included as well.

Our NTA experiments confirmed the size distribution of isolated nanoparticles in the range of 130 nm, which is in agreement with previous findings (Anastasi et al. 2021; Goetzl et al. 2019; Goetzl, Kapogiannis, et al. 2016; Goetzl, Mustapic, et al. 2016). The morphology of these vesicles observed using TEM showed the typical shape of round sac‐like structures in corroboration with the literature (Lim et al. 2019; Théry et al. 2018). To validate the protocol according to the International Society of Extracellular Vesicles, we probed our samples against well‐known EV markers by immunoblotting. Our results confirmed the presence of three external EV markers and two membrane markers, as shown in Figure 4. To confirm the neuronal origin of our isolated fractions, we also identified two neuronal markers (L1CAM and NCAM) via immunoblotting (Goetzl et al. 2018, 2019; Goetzl, Kapogiannis, et al. 2016; Goetzl, Mustapic, et al. 2016; Théry et al. 2018).

After validation of our methodology with three different approaches, we sought to explore the utility of our assay to measure traditional markers of neurodegenerative diseases. The successful measurement of CSF biomarkers such as T‐Tau and P‐Tau in our blood–isolated EV eluates suggests the utility of these preparations as diagnostically feasible. When we compared our results (Figure 5) with the previous findings, the AD EV concentrations of total‐Tau and P‐181‐Tau were similar to previous results (Goetzl et al. 2019). Emerging investigations have shown that exosomal levels of P‐S396‐tau, P‐T181‐tau, and Aβ1–42 in neuronal‐derived exosomes from plasma could predict the progress of AD up to 10 years before clinical onset, showing the biomarker potential of EV‐based assays. A longitudinal study (3–11 years) reported that plasma neuronal exosome levels of P‐181‐Tau, Aβ1‐42, cathepsin D, repressor element 1‐silencing transcription factor, and neurogranin alter with aging in exosomal fractions (Abner et al. 2016). For this work, the researchers isolated exosomes using the ExoQuick kit and immunoprecipitation. In our case, we were able to detect total tau and P‐181‐Tau using our simple nanowire‐based protocol. The successful measurement of CSF biomarkers such as T‐Tau and P‐Tau in our EV eluates suggests the utility of these preparations for biomarker development. There was a limitation of our cohort, so we could not perform an analysis on age‐matched individuals. Future investigations need to explore the biomarker potential of this assay in larger, age‐matched cohorts for generalizability and robustness. Furthermore, baseline characterization of the human plasma samples—including the potential contributions of lipoproteins and platelet‐derived extracellular vesicles (EVs)—was not specifically assessed in our plasma samples. These factors may have influenced the composition and heterogeneity of the samples. Future work will aim to include more comprehensive baseline quality assessments to further strengthen analytical rigor.

The mass spectrometry analysis revealed a distinct proteomic profile of Alzheimer's disease patients, in comparison with healthy controls (Figure 6B). There were 280 proteins with statistical significance when comparing the AD EV‐fractions with healthy controls. These proteins include both upregulated and downregulated proteins. Few examples are displayed in Figure 6A. The CAPNS1 protein (calpain small subunit 1) belongs to the calpain family of potassium‐dependent proteases. The elevated levels of calpain‐1 and cathepsin B activity have an important role in neurodegeneration. The increased abundance of CAPNS1 in AD exosomal fractions suggests an important role of this protein in AD. It has been identified as a potential biomarker (an up‐regulated autophagy gene) for Alzheimer's disease in a bioinformatics study (Li et al. 2023).

The CDC42, a small GTPase, has a significant role in AD by affecting synaptic plasticity and neurodegeneration (Zhu et al. 2023). In agreement with our findings, a reduction in the levels of S100A8 has been validated in plasma extracellular vesicles in AD cases via mass spectrometry and ELISA assays (Zhang et al. 2024). An exosomal proteomics analysis showed the presence of SERPINB12 in plasma EVs in corroboration with our findings (Yao et al. 2024), further validating our EV‐proteomic based findings. The proteomic profiling also highlighted new candidate markers for AD, for example, PSME3 (proteasome activator complex subunit) and FARSA (phenylalanyl‐tRNA synthetase alpha subunit). PSME3 is not currently an established AD biomarker; recent mechanistic studies suggest it plays a role in neurodegenerative processes relevant to AD. It could be considered a promising novel candidate for further research, particularly in the context of immune response (Sun et al. 2016), neuronal senescence, and proteostasis (Yoshioka et al. 2024). Although the candidate proteomic markers were not validated in this exploratory‐phase study, this study has established a valuable resource and methodological foundation for future biomarker discovery efforts. The comprehensive proteomic data generated will facilitate subsequent targeted validation studies and may contribute to the identification of novel candidate biomarkers in larger, independent cohorts. In future studies, we aim to expand our cohort, as well as validate these findings in independent sample sets to further strengthen the evidence for their diagnostic value. Overall, dysregulation (differential abundance) of these proteins (Table S2) in EV fractions and their linkage with Alzheimer's disease pathology demonstrates the successful implementation and significance of the current assay. The simplicity, ease of use, and low cost of the assay make it an attractive and efficient tool for biomarker investigations and clinical implementations.

To recapitulate, in the current study, EV fractions enriched for neuronal EVs were successfully isolated from healthy controls and AD plasma samples using a simple, direct, and innovative technique based on nanotechnology. The EVs enriched for neuronal origin were efficiently extracted from even small sample volumes without the need for additional steps. This technique improves upon traditional methodologies (immunoaffinity‐based) by offering a rapid, scalable, and efficient extraction process.

To conclude, this nanowire‐based protocol is an accurate (cell specific marker‐based), rapid (2 h), and cost‐effective method to isolate neuronal‐derived EVs. The simplicity of the methodology makes it suitable for much‐needed diagnostic utility. Finally, Total‐Tau and P‐Tau (181P‐S199) levels were successfully measured by this method, which represents the successful implementation of this methodology to find out EV‐based early‐stage diagnostic biomarkers from blood plasma. Its compatibility with downstream analyses like ELISA and RT‐QuIC supports the development of liquid biopsies for monitoring disease progression. The presented nanotechnology‐based methodology offers an innovative and efficient tool for EV‐based biomarker investigations and clinical utility by simplifying the enrichment of CNS‐originated EVs from complex biological fluids.

## Author Contributions

**Leticia Camila Fernandez Flores:** conceptualization, writing – original draft, methodology, investigation, writing – review and editing, formal analysis, data curation. **Neelam Younas:** conceptualization, writing – original draft, supervision, writing – review and editing, methodology, investigation, formal analysis, funding acquisition. **Stefan Goebel:** writing – review and editing, formal analysis. **Kathrin Dittmar:** methodology, investigation, writing – review and editing. **Tayyaba Saleem:** methodology, investigation, writing – review and editing. **Abrar Younas:** methodology, investigation, writing – review and editing. **Holger Budde:** methodology, investigation, writing – review and editing, resources. **Tobias J. Legler:** methodology, investigation, writing – review and editing, resources. **Wiebke Möbius:** methodology, investigation, writing – review and editing. **Peter Hermann:** formal analysis, writing – review and editing. **Matthias Schmitz:** writing – review and editing, formal analysis. **Inga Zerr:** conceptualization, supervision, resources, writing – review and editing, writing – original draft, funding acquisition, formal analysis, project administration.

## Ethics Statement

All human subjects provided informed consent. Details are described in the Methods section.

## Conflicts of Interest

The authors declare no conflicts of interest.

## Supporting information


**Data S1:** jnc70263‐sup‐0001‐supinfo.pdf.

## Data Availability

The data that supports the findings of this study is available in the Data S1 of this article.
