# Supplementary material for: Multiple Antibody‐Coated Gold Nanoparticle‐Based ExoAssay for Rapid Isolation of CNS‐Specific Exosomes From Blood
Source: J Neurochem. 2025 Oct 28;169(10):e70263. doi: 10.1111/jnc.70263 (PMC12560209; doi:10.1111/jnc.70263)
Supplement: Supplementary file 1 — Data S1: jnc70263‐sup‐0001‐supinfo.pdf. [file JNC-169-0-s001.pdf]

Multiple antibody-coated gold nanoparticle-based ExoAssay for rapid isolation of CNS-specific exosomes from blood

Leticia Camila Fernandez Flores[1,2#], Neelam Younas[1,2#*], Stefan Goebel[1], Kathrin Dittmar[1], Tayyaba Saleem[1,2], Abrar Younas[1,2], Holger Budde[3], Tobias J. Legler[3], Wiebke Möbius[4], Peter Hermann[1], Matthias Schmitz[1,2], Inga Zerr[1,2]

[1]University Medical Center Göttingen, National Reference Center for Surveillance of TSE, Department of Neurology, Robert-Koch-Strasse 40, 37075 Göttingen, Germany.

[2]German Center for Neurodegenerative Diseases (DZNE), Von-Siebold-Straße 3A, 37075 Göttingen, Germany.

[3]University Medical Center Göttingen, Department of Transfusion Medicine, Robert-Koch Strasse 40, 37075 Göttingen, Germany.

[4]Department of Neurogenetics, Electron Microscopy Unit, Max-Planck-Institute for Multidisciplinary Sciences, Hermann-Rein-Str. 3, 37075 Göttingen, Germany.

shared authorship

*** Correspondence**

Dr. Neelam Younas

National Reference Center
for Surveillance of TSE
Department of Neurology
University of Medicine
Robert-Koch-Strasse 40
37075 Göttingen, Germany
Email: neelam.younas@med.uni-goettingen.de

Tel: +49 (551) 39-65398

Supplementary Material

Table S1: Profiles of the healthy control (HC) participants

Subject	Group	Age	Gender
1	HC	60	Female
2	HC	51	Male
3	HC	59	Female
4	HC	53	Male
5	HC	65	Male
6	HC	56	Female
7	HC	57	Female
8	HC	58	Female
9	HC	58	Male
10	HC	55	Male

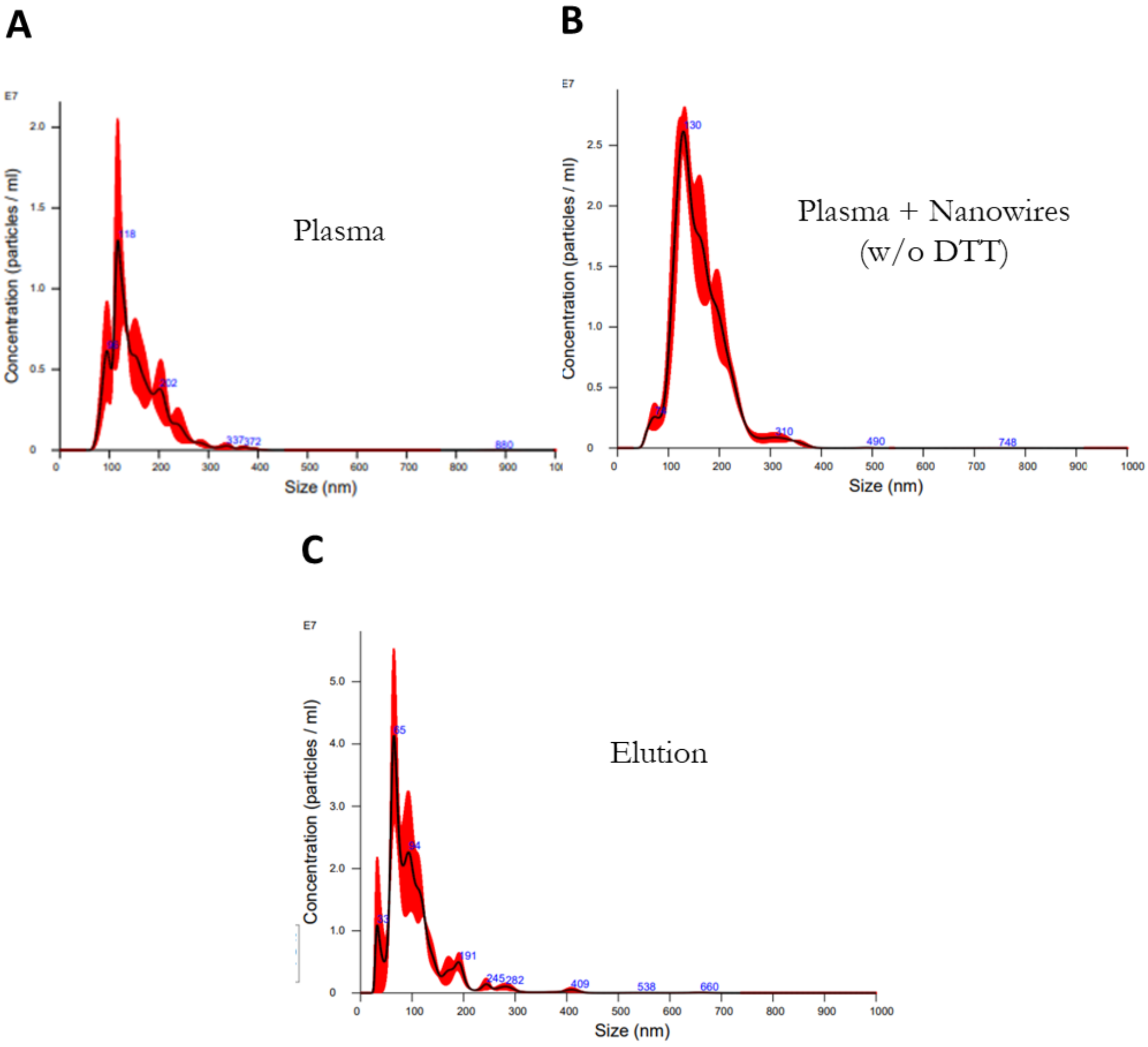

Supplementary Figure 1: NTA results of the distinct fractions of nanowire-based protocol. (A) A single prominent peak of 118 nm with additional small peaks at 97 and 202nm in blood plasma. (B) Plasma with nanowires was denoted by a wide peak of 130 nm. (C) Elution fraction enriched for exosomes, with two peaks 64 nm & 95 nm. Original plasma and the Nanowires bound exosomal fractions were diluted 1:2000 (due to the high concentration of nanoparticles). The elution fraction was diluted at a ratio of 1:1000.

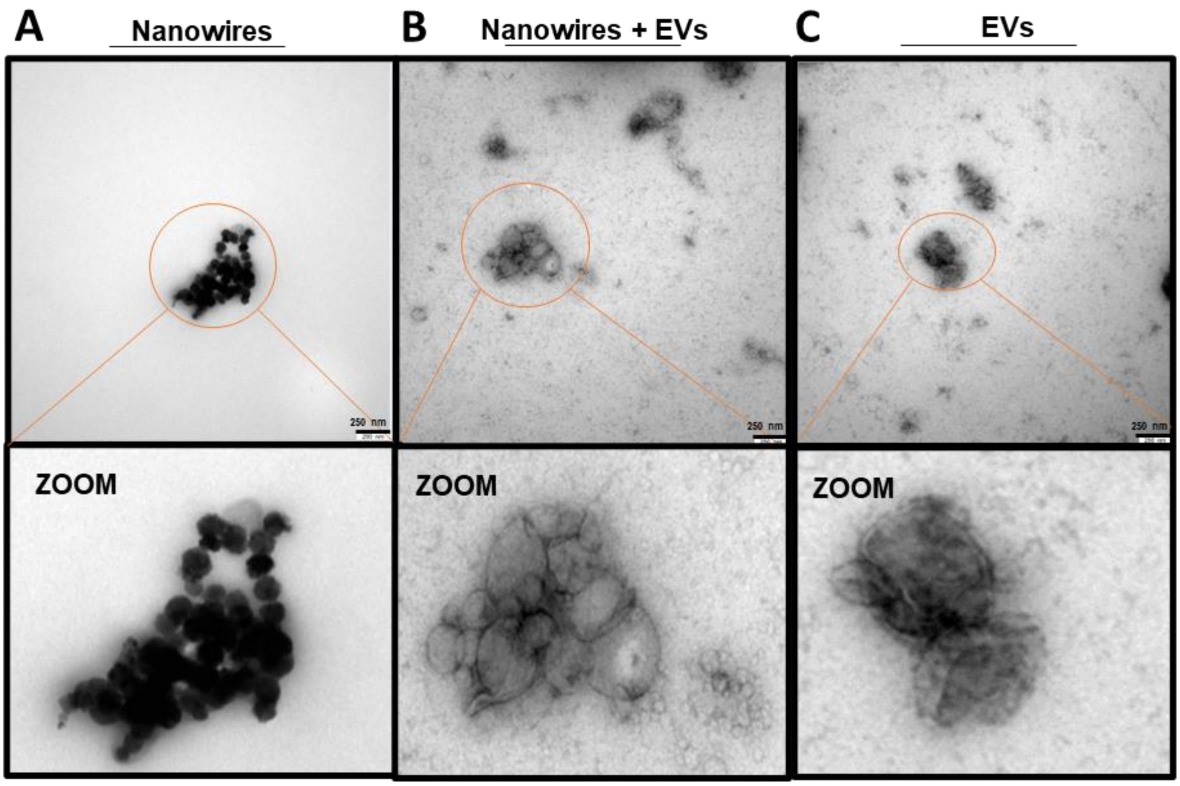

Supplementary Figure 2: Characterization of the EVs by Transmission Electron Microscopy. (A) Nanowires diluted in ddH_2O, which are precipitated. (B) Nanowires are attached to the EVs by antigen-antibody binding. Several EVs can be observed together. (C) EVs released after the addition of 25 mM of DTT. EVs: extracellular vesicles. Bar = 250nm.

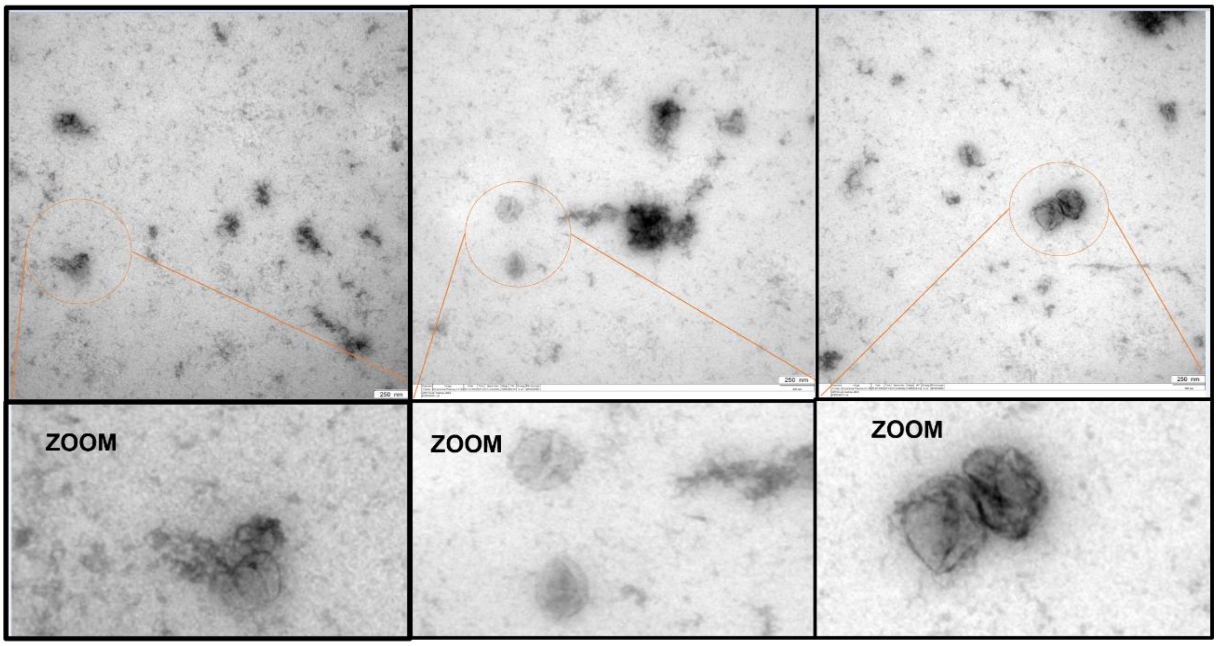

Supplementary Figure 3: Neuronal exosomes isolation final protocol. TEM images of the final protocol eluates in triplicate. Bar = 250nm.

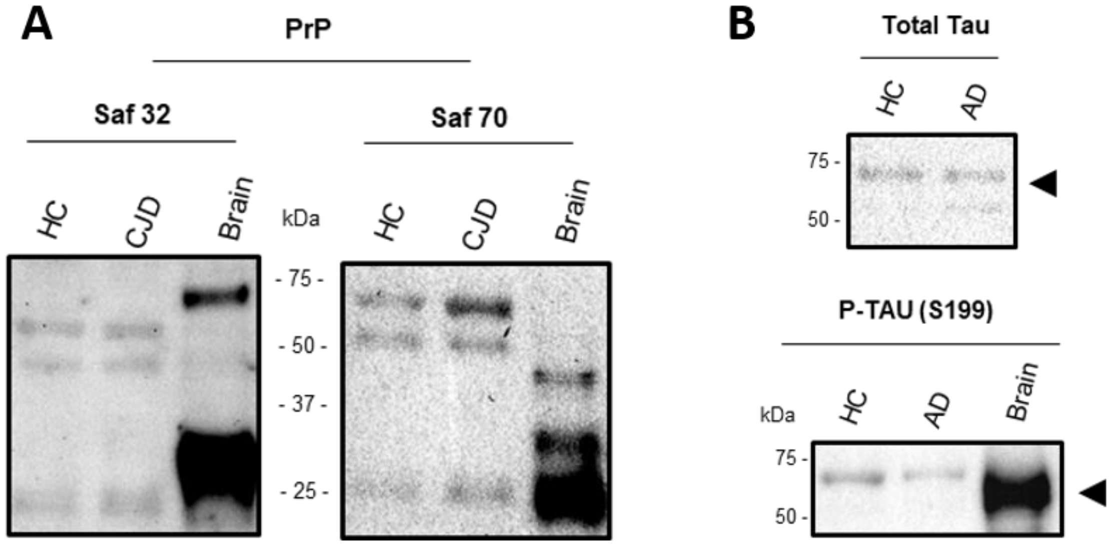

Supplementary Figure 4: Immunoblotting of known neurodegenerative CSF biomarkers in our neuronal-derived EV fractions. (A) HC and CJD neuronal EVs were probed against PrP antibodies (SAF 32 and SAF 70). Both antibodies showed the prion protein band at the expected size. (B) Total Tau and P-Tau (S199) antibodies detected bands in both HC and AD patients. Human brain was used as positive control in the western blots. HC: healthy control (Age: 68Y, Male), AD: Alzheimer's disease (69Y, Male), CJD: Creutzfeldt-Jakob disease (age: 66 Y, Male).

Supplementary Table S2: A list of significantly modified proteins ($p < 0.05$) in EVs isolated from AD patients in comparison with healthy controls with their p-values (-log 10-p-values) and log 2 fold change (FC).

NO.	GENE NAME	PROTEIN DESCRIPTION	P-VALUE	LOG2FC
1	FARSA	Phenylalanine--tRNA ligase alpha subunit (Fragment)	2.39	1.98
2	CAPNS1	Calcium-activated neutral proteinase small subunit	2.81	1.91
3	HEXA	Beta-N-acetylhexosaminidase	1.57	1.69
4	EEF1E1-BLOC1S5	EEF1E1-BLOC1S5 readthrough (NMD candidate)	1.68	1.52
5	CDC42	Cell division control protein 42 homolog	2.03	1.52
6	TRIM25	E3 ubiquitin/ISG15 ligase TRIM25	3.08	1.49
7	ASNS	Asparagine synthetase [glutamine-hydrolyzing]	2.06	1.47
8	GNPNAT1	Glucosamine 6-phosphate N-acetyltransferase (Fragment)	1.87	1.44
9	ENO2	2-phospho-D-glycerate hydro-lyase	1.93	1.44
10	CCAR2	Cell cycle and apoptosis regulator protein 2 (Fragment)	2.06	1.38
11	PDCD11	Programmed cell death 11, isoform CRA_a	2.64	1.38
12	PSME3	Proteasome activator complex subunit 3	2.80	1.36
13	S100A6	Protein S100-A6	2.93	1.32
14	LAP3	Cytosol aminopeptidase	2.14	1.32
15	TRA2B	Transformer-2 protein homolog beta	1.46	1.31
16	MYBBP1A	Myb-binding protein 1A (Fragment)	3.12	1.30

**17**	EIF5	Eukaryotic translation initiation factor 5	2.21	1.30
**18**	SMS	Spermine synthase	2.20	1.27
**19**	SNRPD2	Small nuclear ribonucleoprotein Sm D2	1.68	1.27
**20**	DIS3	Exosome complex exonuclease RRP44	2.32	1.25
**21**	DNAJC7	DnaJ homolog subfamily C member 7	1.81	1.24
**22**	AKR1B1	Aldo-keto reductase family 1 member B1	1.81	1.22
**23**	SLC1A5	Neutral amino acid transporter B(0)	1.97	1.21
**24**	WARS1	Tryptophan--tRNA ligase, cytoplasmic	2.17	1.21
**25**	H4C1	Histone H4	2.17	1.21
**26**	ERP29	Endoplasmic reticulum resident protein 29	2.41	1.21
**27**	TIMM44	Mitochondrial import inner membrane translocase subunit TIM44 (Fragment)	2.15	1.20
**28**	RPL31	60S ribosomal protein L31	1.75	1.19
**29**	CD44	CD44 antigen	2.28	1.18
**30**	BANF1	Barrier-to-autointegration factor	3.75	1.18
**31**	H2BU1	Histone H2B type 3-B	2.06	1.17
**32**	H3-3A	Histone H3	2.85	1.17
**33**	CAPRIN1	Caprin-1 (Fragment)	2.46	1.15
**34**	H2AZ1	Histone H2A	2.13	1.14
**35**	AKR1C3	Aldo-keto reductase family 1 member C3	2.81	1.14
**36**	S100A4	Protein S100-A4	2.36	1.13
**37**	PGK2	Phosphoglycerate kinase 2	2.50	1.13
**38**	NUDT21	Cleavage and polyadenylation specificity factor subunit 5 (Fragment)	1.68	1.12
**39**	NEDD8-MDP1	NEDD8 (Fragment)	2.78	1.11
**40**	SLC16A3	Monocarboxylate transporter 4 (Fragment)	2.57	1.10
**41**	HNRNPH3	Heterogeneous nuclear ribonucleoprotein H3	1.92	1.10
**42**	ALDH9A1	4-trimethylaminobutyraldehyde dehydrogenase	1.99	1.10
**43**	NAA15	N-alpha-acetyltransferase 15, NatA auxiliary subunit	2.08	1.09
**44**	HNRNPA2B1	Heterogeneous nuclear ribonucleoproteins A2/B1	2.27	1.08
**45**	HPCAL1	Hippocalcin-like protein 1	2.26	1.08
**46**	HNRNPR	Heterogeneous nuclear ribonucleoprotein R	2.39	1.07
**47**	PRPF19	Pre-mRNA-processing factor 19	1.31	1.07
**48**	DDX39B	Spliceosome RNA helicase DDX39B (Fragment)	1.92	1.07
**49**	RPS28	40S ribosomal protein S28	2.51	1.07
**50**	TNPO1	Transportin-1	2.53	1.07
**51**	PLIN3	Perilipin-3 (Fragment)	1.79	1.06
**52**	MAGOHB	Protein mago nashi homolog 2	1.92	1.05
**53**	TMCO1	Calcium load-activated calcium channel (Fragment)	2.02	1.04
**54**	TIMM13	Mitochondrial import inner membrane translocase subunit Tim13	1.45	1.04
**55**	UBAP2L	Ubiquitin-associated protein 2-like	2.19	1.04
**56**	HEXB	Beta-N-acetylhexosaminidase (Fragment)	2.01	1.03
**57**	RBMXL1	RNA-binding motif protein, X-linked-like-1 (Fragment)	3.33	1.03

**58**	PRKDC	DNA-dependent protein kinase catalytic subunit	2.44	1.03
**59**	DHX9	ATP-dependent RNA helicase A	2.37	1.02
**60**	H3-2	H3.2 histone (putative) (Fragment)	2.02	1.01
**61**	RAB10	Ras-related protein Rab-10	2.84	1.01
**62**	DSTN	Actin-depolymerizing factor	1.86	1.01
**63**	RPL14	60S ribosomal protein L14	2.07	1.00
**64**	PRPS1	Ribose-phosphate pyrophosphokinase 1	2.30	1.00
**65**	RPLP2	60S acidic ribosomal protein P2	2.19	0.99
**66**	DHX15	Pre-mRNA-splicing factor ATP-dependent RNA helicase DHX15	2.28	0.99
**67**	DUT	Deoxyuridine 5'-triphosphate nucleotidohydrolase	2.82	0.99
**68**	RPL10	60S ribosomal protein L10	1.79	0.99
**69**	TGM2	Protein-glutamine gamma-glutamyltransferase 2	2.73	0.98
**70**	HNRNPA0	Heterogeneous nuclear ribonucleoprotein A0	1.93	0.98
**71**	SEC22B	Vesicle-trafficking protein SEC22b (Fragment)	2.37	0.98
**72**	SERPINB5	Serpin B5	1.91	0.98
**73**	SNRPD3	Small nuclear ribonucleoprotein Sm D3	2.01	0.97
**74**	DDX39A	ATP-dependent RNA helicase DDX39A	1.66	0.97
**75**	MTDH	Protein LYRIC	1.55	0.97
**76**	RPL23A	60S ribosomal protein L23a	1.42	0.97
**77**	YWHAE	14-3-3 protein epsilon	2.06	0.97
**78**	PDIA6	Protein disulfide-isomerase A6	2.31	0.97
**79**	SRSF10	Serine/arginine-rich splicing factor 10	2.10	0.97
**80**	IPO4	Importin-4	1.83	0.96
**81**	UHRF1	RING-type E3 ubiquitin transferase	1.94	0.96
**82**	SSRP1	FACT complex subunit SSRP1	2.37	0.96
**83**	NIBAN2	Protein Niban 2	1.72	0.95
**84**	GRHPR	Glyoxylate reductase/hydroxypyruvate reductase	1.87	0.95
**85**	GTPBP4	Nucleolar GTP-binding protein 1	1.77	0.95
**86**	DDTL	D-dopachrome decarboxylase-like protein	1.99	0.95
**87**	MTHFD1	C-1-tetrahydrofolate synthase, cytoplasmic	2.25	0.95
**88**	EIF5A	Eukaryotic translation initiation factor 5A (Fragment)	1.93	0.94
**89**	KYNU	Kynureninase	1.85	0.94
**90**	SETSIP	Protein SETSIP	2.07	0.94
**91**	HNRNPC	Heterogeneous nuclear ribonucleoproteins C1/C2	2.13	0.94
**92**	H1-10	Histone H1.10	1.88	0.94
**93**	CPS1	Carbamoyl-phosphate synthase [ammonia], mitochondrial	1.92	0.94
**94**	EIF4H	Eukaryotic translation initiation factor 4H	1.83	0.93
**95**	ALDH7A1	Alpha-aminoadipic semialdehyde dehydrogenase	2.02	0.93
**96**	SYNCRIP	Heterogeneous nuclear ribonucleoprotein Q	2.13	0.93
**97**	UBB	Polyubiquitin-B	1.79	0.93
**98**	ASS1	Argininosuccinate synthase	1.81	0.92

**99**	SMAD2	Mothers against decapentaplegic homolog	2.33	0.92
**100**	MRPL12	cDNA FLJ60124, highly similar to Mitochondrial dicarboxylate carrier	1.65	0.91
**101**	S100A11	Protein S100-A11	1.82	0.91
**102**	SRSF3	Serine/arginine-rich-splicing factor 3	2.00	0.91
**103**	SNRPN	Small nuclear ribonucleoprotein-associated protein N (Fragment)	2.16	0.91
**104**	TXN	Thioredoxin	2.38	0.90
**105**	GOLGA2	Golgin subfamily A member 2	1.35	0.90
**106**	XRCC5	X-ray repair cross-complementing protein 5	1.94	0.90
**107**	CAD	Aspartate carbamoyltransferase	1.77	0.90
**108**	PHGDH	D-3-phosphoglycerate dehydrogenase	2.01	0.89
**109**	PGK1	Phosphoglycerate kinase 1	2.35	0.89
**110**	RPS15	40S ribosomal protein S15	1.72	0.89
**111**	DNAJB11	DnaJ_C domain-containing protein (Fragment)	1.85	0.89
**112**	EIF2S3	Eukaryotic translation initiation factor 2 subunit 3	1.44	0.89
**113**	PCBP2	Poly(rC)-binding protein 2 (Fragment)	1.64	0.88
**114**	BOLA2B	BolA-like protein 2	1.39	0.88
**115**	COPG2	Coatomer subunit gamma-2	1.70	0.87
**116**	TM9SF2	Transmembrane 9 superfamily member 2	1.59	0.87
**117**	MCM7	DNA replication licensing factor MCM7	2.26	0.87
**118**	NT5DC1	5'-nucleotidase domain-containing protein 1 (Fragment)	1.34	0.87
**119**	CBR1	Carbonyl reductase [NADPH] 1	1.83	0.87
**120**	*M0QYT0*	RRM domain-containing protein (Fragment)	1.63	0.86
**121**	HPRT1	Hypoxanthine-guanine phosphoribosyltransferase	1.83	0.86
**122**	PRMT1	Protein arginine N-methyltransferase 1 (Fragment)	1.31	0.86
**123**	RPS25	40S ribosomal protein S25	1.34	0.86
**124**	P4HB	Protein disulfide-isomerase	2.22	0.86
**125**	LGALS1	Galectin-1	1.72	0.86
**126**	PICALM	Phosphatidylinositol-binding clathrin assembly protein	1.34	0.85
**127**	LDHA	L-lactate dehydrogenase A chain	1.98	0.85
**128**	EZR	Ezrin	2.39	0.85
**129**	NCL	Nucleolin	1.91	0.84
**130**	EIF3G	Eukaryotic translation initiation factor 3 subunit G (Fragment)	2.14	0.84
**131**	HNRNPM	Heterogeneous nuclear ribonucleoprotein M	2.34	0.84
**132**	HNRNPK	Heterogeneous nuclear ribonucleoprotein K	1.56	0.84
**133**	DEK	Protein DEK	2.06	0.84
**134**	EIF3B	Eukaryotic translation initiation factor 3 subunit B	1.90	0.83
**135**	hCG_2039566	Histone H2A	2.58	0.83
**136**	CSE1L	Exportin-2	1.52	0.83
**137**	NAXE	NAD(P)H-hydrate epimerase	1.73	0.83
**138**	PRDX1	Peroxiredoxin-1 (Fragment)	1.72	0.83
**139**	DDX3X	RNA helicase	2.36	0.81

**140**	RPS12	40S ribosomal protein S12	1.64	0.81
**141**	SUMO3	Small ubiquitin-related modifier 3	1.71	0.81
**142**	FUBP1	Far upstream element-binding protein 1	1.72	0.81
**143**	PRDX3	Thioredoxin-dependent peroxide reductase, mitochondrial	1.52	0.81
**144**	ANP32B	Acidic leucine-rich nuclear phosphoprotein 32 family member B	2.10	0.81
**145**	ECI1	Enoyl-CoA delta isomerase 1, mitochondrial (Fragment)	1.62	0.81
**146**	RNPEP	Aminopeptidase B (Fragment)	1.55	0.80
**147**	ACLY	ATP-citrate synthase	1.83	0.80
**148**	AK2	Nucleoside-diphosphate kinase	2.13	0.80
**149**	RPL13A	60S ribosomal protein L13a	1.89	0.80
**150**	FASN	3-hydroxyacyl-[acyl-carrier-protein] dehydratase	1.86	0.80
**151**	PLS3	Plastin-3	1.73	0.80
**152**	EIF3D	Eukaryotic translation initiation factor 3 subunit D	2.09	0.79
**153**	AP1B1	AP-1 complex subunit beta-1	1.64	0.79
**154**	PPIA	Peptidyl-prolyl cis-trans isomerase A	2.17	0.79
**155**	CALR	Calreticulin	1.84	0.79
**156**	ACTG1	Actin, cytoplasmic 2 (Fragment)	2.72	0.79
**157**	OTUB1	Ubiquitinyl hydrolase 1	1.66	0.79
**158**	MCM6	DNA replication licensing factor MCM6	1.43	0.78
**159**	BAG2	BAG family molecular chaperone regulator 2	1.78	0.78
**160**	RPN2	Dolichyl-diphosphooligosaccharide--protein glycosyltransferase subunit 2	1.98	0.77
**161**	H2BC12	Histone H2B type 1-K	2.31	0.77
**162**	C1QBP	Complement component 1 Q subcomponent-binding protein, mitochondrial	1.69	0.77
**163**	SUPT16H	FACT complex subunit SPT16	2.93	0.76
**164**	SNRPE	Small nuclear ribonucleoprotein E	1.93	0.76
**165**	PABPC1	Polyadenylate-binding protein	1.76	0.76
**166**	PABPN1	Polyadenylate-binding protein 2	2.31	0.75
**167**	KPNB1	Importin subunit beta-1	1.68	0.75
**168**	TPM3	Tropomyosin alpha-3 chain	1.97	0.75
**169**	MCM4	DNA helicase (Fragment)	2.76	0.75
**170**	RPS10	40S ribosomal protein S10	1.51	0.74
**171**	MIF	Macrophage migration inhibitory factor	2.05	0.74
**172**	HNRNPU	Heterogeneous nuclear ribonucleoprotein U (Fragment)	1.62	0.74
**173**	RPS24	40S ribosomal protein S24	1.53	0.74
**174**	RPLP0	60S acidic ribosomal protein P0	1.47	0.73
**175**	COPB1	Coatomer subunit beta (Fragment)	1.40	0.73
**176**	CSTB	Cystatin-B	2.12	0.72
**177**	RAN	GTP-binding nuclear protein Ran	1.38	0.72
**178**	MCM5	DNA helicase	1.71	0.72
**179**	RPL12	60S ribosomal protein L12	2.05	0.72
**180**	APOC1	Apolipoprotein C-I (Fragment)	2.28	0.72
**181**	EIF3F	Eukaryotic translation initiation factor 3 subunit F	1.75	0.72

**182**	HNRNPH1	Heterogeneous nuclear ribonucleoprotein H	2.41	0.72
**183**	CCT2	T-complex protein 1 subunit beta	2.25	0.71
**184**	EIF4G1	Eukaryotic translation initiation factor 4 gamma 1	2.33	0.71
**185**	EIF4A1	Eukaryotic initiation factor 4A-I	1.79	0.71
**186**	XRCC6	X-ray repair cross-complementing protein 6	1.56	0.71
**187**	ANXA2	Annexin A2	1.57	0.71
**188**	TMEM33	Transmembrane protein 33 (Fragment)	1.57	0.70
**189**	FKBP3	Peptidyl-prolyl cis-trans isomerase FKBP3	1.39	0.70
**190**	RPS14	40S ribosomal protein S14 (Fragment)	1.73	0.70
**191**	PPIB	Peptidyl-prolyl cis-trans isomerase B	2.69	0.70
**192**	ARCN1	Coatomer subunit delta	1.81	0.69
**193**	HNRNPF	Heterogeneous nuclear ribonucleoprotein F	1.69	0.68
**194**	GANAB	Neutral alpha-glucosidase AB	1.79	0.68
**195**	VIM	Vimentin	1.77	0.68
**196**	CKB	Creatine kinase B-type	1.72	0.68
**197**	HLA-A	HLA class I histocompatibility antigen, A alpha chain	1.87	0.67
**198**	KHSRP	Far upstream element-binding protein 2	1.61	0.66
**199**	PRDX6	Peroxiredoxin-6	1.71	0.66
**200**	PDIA3	Protein disulfide-isomerase A3 (Fragment)	1.55	0.65
**201**	ANXA5	Annexin A5	1.75	0.65
**202**	DDX1	ATP-dependent RNA helicase DDX1	1.34	0.64
**203**	SOD1	Superoxide dismutase [Cu-Zn]	1.46	0.64
**204**	RPL11	60S ribosomal protein L11	1.41	0.63
**205**	PCBP1	Poly(rC)-binding protein 1	1.69	0.62
**206**	APOC4	Apolipoprotein C-IV	1.53	0.62
**207**	MCM3	DNA replication licensing factor MCM3	1.31	0.62
**208**	DDX5	DEAD box protein 5	1.48	0.61
**209**	NPEPPS	Aminopeptidase	1.36	0.61
**210**	ENO1	Alpha-enolase	1.53	0.61
**211**	UBE2N	Ubiquitin-conjugating enzyme E2 N	1.41	0.60
**212**	CST3	Cystatin-C	1.59	0.60
**213**	IQGAP1	Ras GTPase-activating-like protein IQGAP1	1.34	0.60
**214**	SFPQ	Splicing factor, proline- and glutamine-rich	1.32	0.59
**215**	CCT8	T-complex protein 1 subunit theta	1.74	0.59
**216**	ACTN4	Alpha-actinin-4	1.71	0.59
**217**	LMNA	Prelamin-A/C	1.38	0.59
**218**	CFL1	Cofilin-1	1.47	0.58
**219**	LGALS3	Galectin-3	1.36	0.57
**220**	RPS21	40S ribosomal protein S21	1.44	0.57
**221**	ANXA3	Annexin	1.43	0.57
**222**	SEPTIN2	Septin-2	2.00	0.56
**223**	MATR3	Matrin-3	1.39	0.56
**224**	RPS7	40S ribosomal protein S7	1.75	0.55
**225**	BLVRB	Flavin reductase (NADPH)	1.97	0.55
**226**	GDI2	Rab GDP dissociation inhibitor beta	1.54	0.54
**227**	ARF3	ADP-ribosylation factor 3	1.59	0.53

**228**	HNRNPD	Heterogeneous nuclear ribonucleoprotein D0 (Fragment)	1.90	0.53
**229**	PNP	Purine nucleoside phosphorylase	1.87	0.52
**230**	CCT3	T-complex protein 1 subunit gamma	1.65	0.52
**231**	ILF3	Interleukin enhancer-binding factor 3	1.33	0.51
**232**	CYB5R3	NADH-cytochrome b5 reductase 3	1.42	0.48
**233**	CCT4	T-complex protein 1 subunit delta	1.94	0.48
**234**	NPM1	Nucleophosmin	1.34	0.46
**235**	TFRC	Transferrin receptor protein 1	1.45	0.45
**236**	CCT6A	T-complex protein 1 subunit zeta	1.55	0.45
**237**	NANS	Sialic acid synthase	1.37	0.44
**238**	ANXA1	Annexin A1	1.34	0.42
**239**	TAGLN2	Transgelin-2	1.40	0.42
**240**	APOD	Apolipoprotein D (Fragment)	1.49	0.41
**241**	RPN1	Dolichyl-diphosphooligosaccharide--protein glycosyltransferase subunit 1	1.40	0.40
**242**	MAST2	Non-specific serine/threonine protein kinase	1.35	0.38
**243**	TKT	Transketolase	1.47	0.30
**244**	AHSG	Alpha-2-HS-glycoprotein	1.40	-0.39
**245**	PLG	Plasminogen	1.39	-0.40
**246**	CPB2	Carboxypeptidase B2	1.31	-0.42
**247**	APOM	Apolipoprotein M	1.44	-0.44
**248**	SELENO P	Selenoprotein P (Fragment)	1.36	-0.49
**249**	TF	Serotransferrin	1.77	-0.52
**250**	PON1	Serum paraoxonase/arylesterase 1	1.45	-0.52
**251**	HABP2	Hyaluronan-binding protein 2	1.41	-0.53
**252**	SERPINA 10	Protein Z-dependent protease inhibitor	1.34	-0.55
**253**	BTD	Biotinidase	1.36	-0.58
**254**	C4B	C4a anaphylatoxin (Fragment)	1.42	-0.67
**255**	PROZ	Vitamin K-dependent protein Z	1.34	-0.70
**256**	MMP9	Matrix metalloproteinase-9	1.39	-0.72
**257**	GPX3	Glutathione peroxidase	1.87	-0.77
**258**	PPBP	Platelet basic protein	1.58	-0.77
**259**	CSTA	Cystatin-A	1.44	-0.78
**260**	PIP	Prolactin-inducible protein	1.64	-0.82
**261**	IL1RAP	Interleukin-1 receptor accessory protein (Fragment)	1.58	-0.83
**262**	THBS1	Thrombospondin-1	1.94	-0.86
**263**	MASP1	Mannan-binding lectin serine protease 1	1.50	-0.87
**264**	S100A9	Protein S100-A9	1.39	-0.88
**265**	FN1	Isoform 1 of Fibronectin	1.55	-0.91
**266**	ACTR2	Actin-related protein 2	2.56	-0.94
**267**	DSG1	Desmoglein-1	1.68	-0.95
**268**	IGHV3-38	Probable non-functional immunoglobulin heavy variable 3-38	2.72	-0.96
**269**	CNDP1	Beta-Ala-His dipeptidase	1.60	-1.00
**270**	ITGB3	Integrin beta-3	1.37	-1.03
**271**	FBLN1	Fibulin-1	1.66	-1.08

**272**	MMRN1	Multimerin-1	1.72	-1.10
**273**	GP1BA	Glycoprotein Ib (Platelet), alpha polypeptide	2.00	-1.10
**274**	CRTAC1	Cartilage acidic protein 1	1.32	-1.16
**275**	LCP1	Plastin-2	2.14	-1.19
**276**	STOM	Stomatin	2.59	-1.42
**277**	MYADM	Myeloid-associated differentiation marker (Fragment)	1.88	-1.49
**278**	S100A8	Protein S100-A8	2.58	-1.61
**279**	RAB27B	Ras-related protein Rab-27B	1.77	-1.78
**280**	SERPINB 12	Serpin B12	2.47	-1.94

66
